# Comparison of brain serotonin transporter using [I-123]-ADAM between obese and non-obese young adults without an eating disorder

**DOI:** 10.1371/journal.pone.0170886

**Published:** 2017-02-09

**Authors:** Chih-Hsing Wu, Chin-Sung Chang, Yen Kuang Yang, Lie-Hang Shen, Wei-Jen Yao

**Affiliations:** 1 Departments of Family Medicine, National Cheng Kung University College of Medicine and Hospital, Tainan, Taiwan; 2 Institutes of Behavioral Medicine, National Cheng Kung University College of Medicine, Tainan, Taiwan; 3 Institutes of Gerontology, National Cheng Kung University College of Medicine, Tainan, Taiwan; 4 Institutes of Clinical Medicine, National Cheng Kung University College of Medicine, Tainan, Taiwan; 5 Departments of Psychiatry, National Cheng Kung University College of Medicine and Hospital, Tainan, Taiwan; 6 Institute of Nuclear Energy Research, Longtan Township, Taoyuan County, Taiwan; 7 Departments of Nuclear Medicine, National Cheng Kung University College of Medicine and Hospital, Tainan, Taiwan; University of Barcelona, Faculty of Biology, SPAIN

## Abstract

Cerebral serotonin metabolism has an important but controversial role in obesity. However, it is not given enough attention in morbidly obese young adults. We used single photon emission computed tomography (SPECT) with [I-123]-labeled 2-((2-((dimethylamino)methyl)phenyl)thio)-5-iodophenylamine (ADAM) to investigate changes in serotonin transporter (SERT) availability in 10 morbidly obese young adults without an eating disorder (M/F = 5/5, body mass index (BMI): 40.3 ± 4.1 kg/m^2^, percentage of body fat (BF%): 46.0 ± 3.9%) and 10 age- and sex-matched non-obese controls (BMI: 20.3 ± 1.2 kg/m^2^, BF%: 20.6 ± 8.9%). All participants underwent SPECT at 10 min and 6 h after an injection of 200 MBq of [I-123]-ADAM. The SERT binding site (midbrain) was drawn with cerebellum normalization. The BF% and fat distribution were measured using dual-energy X-ray absorptiometry. The midbrain/cerebellum SERT binding ratios (2.49 ± 0.46 vs. 2.47 ± 0.47; *p* = 0.912) at 6 h were not significantly different between groups, nor was the distribution of the summed images at 10 min (1.36 ± 0.14 vs. 1.35 ± 0.11; *p* = 0.853). There were no significant correlations between midbrain/cerebellum SERT binding ratio and age, BMI, BF%, or fat distribution. No significant difference in SERT availability in the midbrain between morbidly obese and non-obese young adults without an eating disorder indicates an unmet need for investigating the role of cerebral serotonin in obesity.

## Introduction

Obesity and its associated comorbidities, e.g., cardiovascular disease, diabetes, inflammatory diseases, cancer, and mortality, are an ongoing healthcare problem in Asia [1] and worldwide [2]. In the USA, about 4.2% of young men and 7.6% of young women between 20 and 39 years old are morbidly obese [3]. However, long-term comorbidities and management strategies for obesity have rarely been studied in young adults [4].

Obesity is generally considered a key factor for developing metabolic syndrome and cardiovascular events [5]. The diverse theories of pathophysiological mechanisms for obesity might explain its broad variation of clinical presentations and its varied responses to different interventions. Of greatest interest is the brain and satiety control. Many neuropeptides (such as α-melanocyte-stimulating hormone [α-MSH], neuropeptide Y, agouti-related peptide [AGRP], etc.) are released centrally and act on other neurons at specific receptors to regulate food intake [6]. Frequently, the discussions of the clinical management of obesity focus on the regulation of food intake by dopamine and serotonin receptors in the brain [7]. However, the dopamine D2 receptors and dopamine transporter (DAT) in obese people are not consistently proportional to their body mass index (BMI) [8–10]. Serotonin (5-HT) is one of the key neurotransmitters or neuromodulators involved in fundamental cerebral functions and behaviors, such as sleep, appetite, moods, and emotions [11]. Recently, new anti-obesity drugs (lorcaserin, bupropion HCl/naltrexone HCl, phentermine/topiramate extended-release, etc.) that target neurotransmitters or neuropeptides have been developed and US FDA-approved, but the inconsistent effects that reflecting the complicated mechanism have not been adequately addressed [6]. It is of particular interest that the central serotonergic activities have been the most discussed in recent debates [11].

The availability of serotonin transporter (SERT) was assessed using single photon emission computed tomography (SPECT) with [I-123]-ADAM 2-((2-((dimethylamino)methyl)phenyl)thio)-5-iodophenylamine, a radioligand confirmed to have a high selectivity for imaging SERT availability in SPECT [12,13]. We previously showed [14] the test-retest reproducibility of [I-123]-ADAM for different durations and reconfirmed [15] the correlation between [I-123]-ADAM-derived SERT activity and somatization symptoms. Only a few studies focus on the association between SERT activity and obesity in patients with an eating disorder. A significantly lower level of brain SERT was reported [16] in 11 obese binge-eating women than in 7 obese non-binge-eating middle-aged women. One twin study [17] reported that 9 lifetime purging bulimic women had higher SERT binding in the midbrain, but not in the thalamus or cerebellum, than did 24 healthy women. These reports might reveal the importance of serotonin in obese people with an eating disorder. However, there are some conflicting findings about SERT binding in people without an eating disorder [18,19]. Because people with an eating disorder comprise only 10–20% of the obese population [20–22], it would be more interesting to know the role of SERT binding in obese people without an eating disorder. Furthermore, because the effect of neurotransmitters on body weight might be confounded by environmental factors and aging [23], it should be less confounded to study these interrelationships in relatively young people. Our literature review showed no published investigations of SERT binding in morbidly obese young adults without an eating disorder. Therefore, we investigated specific [I-123]-ADAM binding to brain SERT in a living brain to determine whether there are quantitative changes of SERT between healthy non-obese and morbidly obese young adults without an eating disorder.

## Materials and methods

### Study participants

Ten morbidly obese young people (M/F = 5/5; mean age: 24.8 ± 4.9 years; mean BMI: 40.3 ± 4.1 kg/m^2^) were recruited from the programmed weight management outpatient clinic in a tertiary care medical center [24]. Exclusion criteria were a history of psychiatric or neurological disease, head trauma with loss of consciousness for more than 30 min, undertreated hypertension, diabetes, or medical conditions that might have altered their cerebral functioning, using anorexic medications or surgical procedures for weight loss in the previous 6 months, using any systemic medications in the previous 4 weeks, smoking or alcohol or other substance abuse, participating in a trial for weight control in the previous 12 months, and a high risk of eating disorders based on SCOFF questionnaires (a screening tool for eating disorders) [25]. Ten age- and sex-matched non-obese healthy volunteers (mean age: 22.8 ± 1.6 years; mean BMI: 20.3 ± 1.2 kg/m^2^) were enrolled as the control group. This study was approved by the Institutional Review Board of National Cheng Kung University Hospital (IRB: NCKUH-92-21), and all participants provided written informed consent before the study began.

### Procedures

#### [I-123]-ADAM SPECT imaging

After an overnight fast, each participant was injected with 185 MBq (5 mCi) of [I-123]-ADAM (provided by the Taiwan Institute of Nuclear Energy Research). The radioligand was slowly injected into the antecubital vein in a dimly lit and quiet room. Early and delayed SPECT scans (10 min and 6 h, respectively, post-injection) were done using a dedicated MultiSPECT 3 gamma camera with fan-beam collimators (Siemens Medical Systems; Hoffman Estates, IL, USA) [26]. The acquisition parameters consisted of 120 projection angles over 360 degrees, and a 128 × 128 matrix with a pixel-width of 2.9 mm in the projection domain. A 20% energy window was symmetrically centered at 159 keV. Data collection lasted for approximately 20 min (30 s/projection). The projection images were reconstructed using filtered back-projection with a Butterworth and Ramp filter (cutoff frequency = 0.3 Nyquist; power factor = 7). For uniform attenuation correction, Chang’s first order method was used [27]. The SPECT images were registered to a magnetic resonance image (MRI) using a validated two-step method (at 10 min and at 6 h) [26]. The 10-min ADAM SPECT image is similar to a hexa-methyl-propylene-amine-oxime (HMPAO) SPECT image, which might provide the relative distribution of blood flow, and which allows reliable registration to MRIs using the two-step method. Because the midbrain area has the highest concentration of SERT binding sites [28], the midbrain, as the specific binding site, and the cerebellum, as the non-specific site where serotonin transporters are lacking, were both drawn on MRIs [29]. Pseudo-colors of brain images were used to reflect the highest SERT concentration as white and the lowest as blue [26]. For semi-quantification, the SERT of the midbrain region was normalized to the cerebellum and calculated as a midbrain/cerebellum (MID/CE) ratio [29]. A modified version of Statistical Parametric Mapping 99 (MathWorks, Natick, MA, USA) was used for group comparison.

#### Anthropometry and Dual-energy X-ray Absorptiometry (DXA)

Each participant’s body height and weight were measured using a balanced scale (Detecto, Webb City, MO, USA), and their BMIs were calculated. Before the [I-123]-ADAM SPECT examination day, the whole body composition of each participant was measured using dual-energy X-ray absorptiometry (DXA) (DPX-L with 1.3 z; Lunar Radiation, Madison, WI, USA). In our lab, we enrolled 128 participants to calculate the coefficients of variation (CVs) in their DXA scans. The CVs for replicate measurements were 1.0% for total lean tissue mass and 2.9% for total fat tissue mass [30]. The percentage of body fat (BF%) derived using DXA was calculated as a ratio of [total body fat mass]/[total body weight] [30]. The body fat distribution, reflected by the percentages of trunk and leg fat, was calculated as regional fat divided by regional body mass [30]. For obese participants, the BF% was defined as ≥ 38% in females and ≥ 25% in males [31].

### Statistical analysis

SPSS 13 for Windows (SPSS Inc., Chicago, IL, USA) was used for all analyses. The outcome measurements were the ratios of specific to nonspecific [I-123]-ADAM binding (midbrain/cerebellum), body weight, BMI, BF%, and fat distribution. The results are given as means ± standard deviation (SD). Differences between non-obese and morbidly obese participants were tested. Continuous variables were analyzed using a nonparametric Mann-Whitney test. Categorized variables were analyzed using a χ^2^ test with a Fisher’s exact test and the nonparametric McNemar-Bowker test of symmetry. The relationship between the parametric factors and the ratios of specific-to-nonspecific binding in [I-123]-ADAM SPECT (midbrain/cerebellum ratio) were examined using the Spearman rank correlation test. Significance was set at *p* < 0.05 (two-tailed).

## Results

**Fig 1:**
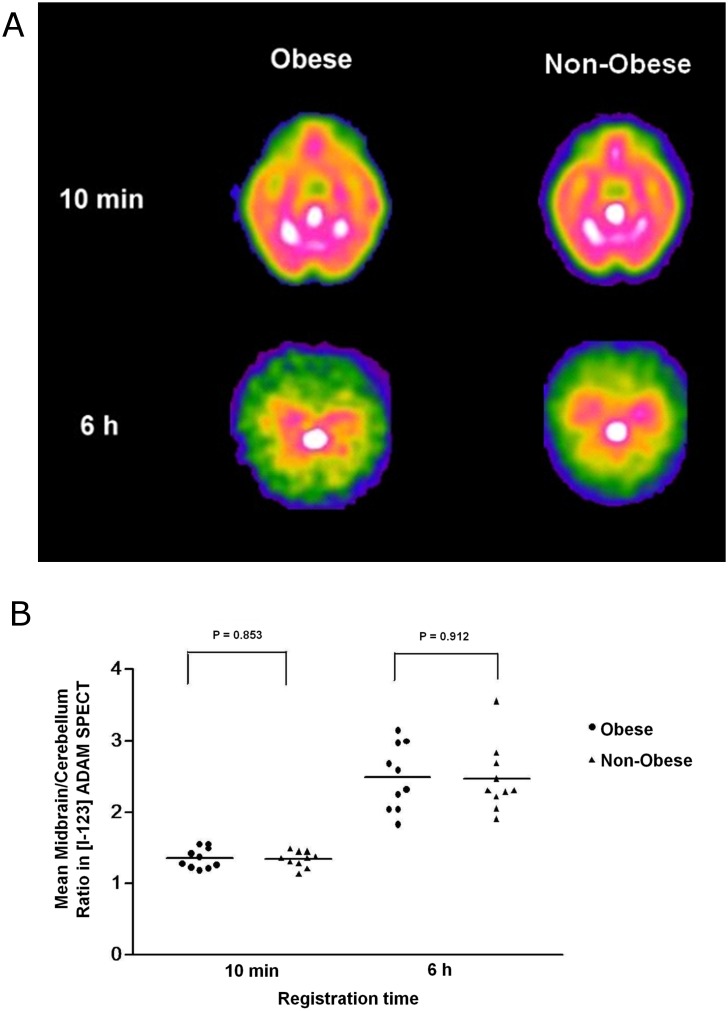
(a) Summed images of [I-123]-ADAM SPECT at midbrain levels of members of the morbidly obese and of the non-obese groups at 10 min and at 6 h after the injection of the radioligand. The regions of interest of the midbrain are in white in all images. (b) There were no significant differences in radioligand delivery at 10 min (1.36 ± 0.14 vs. 1.35 ± 0.11; p = 0.853) or in the midbrain/cerebellum (MID/CE) SERT binding ratio at 6 h (2.49 ± 0.46 vs. 2.47 ± 0.47; p = 0.912) between the obese (●) and non-obese (▲) groups.

Except for the BMIs, BF%, and fat distribution, there were no significant differences in sex or age distribution between non-obese and morbidly obese participants (Table 1). Ten minutes post-injection, the distribution of [I-123]-ADAM in the brain was similar between groups (1.36 ± 0.14 vs. 1.35 ± 0.11, p = 0.853) which reflected their comparable brain blood-flow distribution status. Six hours post-injection, the distribution of SERT was highest in the midbrain and lowest in the cerebellum (Fig 1a). The midbrain/cerebellum ratio ranges were: morbidly obese group = 1.91–3.56, and non-obese group = 1.83–3.15. The mean midbrain/cerebellum ratio did not significantly differ by weight groups (2.49 ± 0.46 vs. 2.47 ± 0.47, respectively; p = 0.912) (Fig 1b) or by sex (S1 Table). The scatter plots (S1 and S2 Figs) and correlations between the midbrain/cerebellum ratios and age, BMI, BF%, trunk fat%, and leg fat% were not significantly different at 10 min or 6 h after the SPECT scan (Table 2).

**Table 1:** Clinical characteristics of the obese and non-obese groups.

	Morbidly Obese	Non-Obese	*P*-value^†^
Cases (n)	10	10	
Gender (M:F)	5:5	5:5	1.000
Age (years)	24.8 ± 4.9	22.8 ± 1.6	0.684
Range	21–35	20–26	
Body height (cm)	166.1 ± 5.7	167.4 ± 5.3	0.631
Range	158.0–174.0	159.0–179.0	
Body weight (kg)	111.1 ± 12.4	57.1 ± 4.8	< 0.001
Range	92.0–127.0	52.0–65.0	
Body mass index (kg/m^2^)	40.3 ± 4.1	20.3 ± 1.2	< 0.001
Range	34.4–46.1	19.3–22.8	
Percentage of body fat (%)^‡^	46.0 ± 3.9	20.6 ± 8.9	< 0.001
Trunk fat (%)^‡^	45.4 ± 4.1	20.1 ± 8.2	< 0.001
Leg fat (%)^‡^	47.2 ± 4.6	22.5 ± 10.5	< 0.001

Data are mean ± SD unless otherwise specified.

^†^Mann-Whitney test for continuous variables, χ^2^ test for gender difference.

^‡^ The fat percentages were all derived using dual-energy X-ray absorptiometry (DXA).

**Table 2:** Correlation coefficients^†^ between the distribution of midbrain/cerebellum ratios (MID/CE) in [I-123]-ADAM SPECT image and body composition in 20 morbidly obese and non-obese young adults.

	MID/CE (6 h)	Age (years)	BMI (kg/m^2^)	PBF (%)	Trunk Fat (%)	Leg Fat (%)
MID/CE (10 min)	0.212	0.076	0.123	0.394	0.384	0.415
MID/CE (6 h)	-	0.178	0.123	0.196	0.264	0.123

BMI, body mass index (kg/m^2^); PBF, percentage of body fat.

^†^ Spearman rank correlation test, no statistical significance.

## Discussion

We found no significant correlations between midbrain serotonin transporter availability and BMI or BF%. Consequently, we found no significant differences in radiotracer delivery (reflected by the 10-min MID/CE ratio) and SERT availability (reflected by the 6-h MID/CE ratio) between morbidly obese and non-obese patients without an eating disorder. The findings were consistent in participants when analyzed in total and when stratified by sex (S1 Table).

The serotonergic system is associated with controlling the body’s energy balance [32] and with gut-brain interactions [33] that regulate body weight [34]. Several studies [35,36] have found that serotonin is involved in controlling eating behavior and body weight in humans. Patients with eating disorders might have either lower [16] or higher [17] SERT binding in the mid-brain than do healthy controls. Because nearly 20% of obese patients have a substantial eating disorder [20–22], understanding the serotonergic activity in the regulation of obesity is of value. The SCOFF questionnaires [25] have been validated for screening eating disorders. Setting the threshold at two or more positive answers to all five questions provided 92.6–100% sensitivity and 79.2%-93.4% specificity [25]. Our study participants were also screened using SCOFF questionnaires and none had a score ≥ 2 positive answers. Because our study participants had no eating disorders, that there was no difference in SERT binding between the morbidly obese and non-obese groups might reflect a different serotonin pathway in obese young adults without an eating disorder.

The lower SERT immunoreactivity in the infundibular nucleus of obese participants [37], and the downregulation of SERT in the platelet membranes of morbidly obese participants [38], indicate the involvement of 5-HT in human obesity. Increased food intake (e.g., snacking) might significantly decrease (by 30%) SERT-binding [39]. In contrast, 5-HT activity might upregulate hunger and food intake [35,40]. Using an [11C]DASB PET scan with MR imaging, to obtain a more accurate region, showed an inverse relationship between cerebral SERT and BMI in middle-aged volunteers [19]. Whether there is a positive correlation between SPECT-derived SERT and BMI in the thalamus across 20- to 90-year-olds [41] or within twins [18] has not been confirmed, but there are no differences in the midbrain [41] or in young and non-obese people [18]. Because our participants were relatively young and were neither binge eaters nor bulimic, the confounding effect of aging and an eating disorder might have been reduced. Furthermore, the MID/CE ratio of the SERT binding site was measured in our study, not the thalamus/hypothalamus ratio [18, 41] or midbrain/brainstem ratio [41], which might have yielded different results. There was also no significant correlation between SERT availability and the duration of exposure to sunlight [27] or between SERT availability and exercise.

In contrast, serotonergic activity is known to have an anorexic effect, which has been observed through the agonism of the 5-HT_1B_ and 5-HT_2C_ receptors, and the antagonism of the 5-HT_6_ receptor [42]. Although lorcaserin (APD-356, a selective 5-HT_2C_ agonist) has been approved worldwide for treating adults with a BMI equal to or greater than 30, and adults with a BMI of 27 or greater who have at least one weight-related health condition, such as high blood pressure, type 2 diabetes, or high cholesterol [43–46], the inconsistent response to these serotonergic drugs [47] might indicate the wide variation of individual serotonergic activity and the importance of non-serotonergic pathophysiology in obesity. Therefore, it is plausible to find a non-significant difference in SERT binding between morbidly obese and non-obese young adults without an eating disorder.

Based on a 3-compartment model, DXA is a well-validated method for measuring body composition and fat distribution [30]. The correlations between SERT and DXA-derived body-fat percentage and fat distribution have never been well studied. In our cross-sectional study, we found a relatively high but nonsignificant correlation in our young adults. The clinical implications of these unique findings merit further evaluation.

This study has several limitations. First, our limited number of cases might weaken the importance of our findings. Other studies [16–18], with 7 to 25 cases, however, show the changes in SERT binding. Because SERT availability was much closer between the non-obese and morbidly obese groups, our findings will be consistent, even with more participants. Second, we did not enroll overweight and non-morbidly obese participants: if there is no difference between the two ends (the non-obese and the morbidly obese), it should be difficult to find any significant differences between the two groups in the middle. Third, at the time of imaging, no intravenous blood sample were taken to confirm the equilibrium between brain regions and blood plasma with respect to ADAM. Fourth, using SPECT to detect midbrain SERT represent a problem of resolution. There are multiple serotonergic nuclei in the midbrain that might express pleiotropic properties through distinct regulation of SERT in diverse serotonergic populations. If SERT did change in a specific population in response to obesity, such a change could be obscured within a measure of multiple serotonergic populations. Therefore, SERT measured in the serotonergic nuclei might not equal SERT regulation in the axons. Using an MRI image as a reference to define the corresponding anatomical region might provide a more accurate “midbrain” area than does using SPECT [48]. Furthermore, we focused on the SERT level in the midbrain because of the high availability of SERT in the midbrain [49] and because [I-123]-ADAM SPECT is an ideal tracer for imaging SERT in the midbrain, but not in the striatum or thalamus because of its low signal-to-noise ratios [50]. Except for the midbrain regions, it would also be better to understand the other brain region-specific changes, e.g., terminal region changes, to disclose the whole spectrum of serotonergic activities. Finally, regulating body weight in obese people without an eating disorder is more complex and might involve other physiological mechanisms and other neurotransmitters [23], particularly the brain dopaminergic and noradrenergic systems as well as the leptin receptor (OB-R), which are not assessed in this study but should be concomitantly studied in future research.

## Conclusion

The lack of a significant difference in SERT availability in the midbrain between morbidly obese and non-obese young adults without an eating disorder indicates the complexity of the neurobiological pathophysiology of obesity. The serotonergic approach of weight management in obese young adults warrants more comprehensive study in the future.

## Supporting information

S1 Fig Scatter plots of correlation coefficients (from (a) to (f)) between the distribution of 10 min midbrain/cerebellum ratios (MID/CE) in [I-123]-ADAM SPECT image, age and body composition in 20 morbidly obese and non-obese young adults. (DOC)

S2 Fig Scatter plots of correlation coefficients (from (a) to (f)) between the distribution of 6h midbrain/cerebellum ratios (MID/CE) in [I-123]-ADAM SPECT image, age and body composition in 20 morbidly obese and non-obese young adults. (DOC)

S1 Table Comparisons of SERT in midbrain/cerebellum (MID/CE) ratios between obese and non-obese young adults stratified by sex. (DOC)
