# Supplementary material for: Comparison of brain serotonin transporter using [I-123]-ADAM between obese and non-obese young adults without an eating disorder
Source: PLoS One. 2017 Feb 9;12(2):e0170886. doi: 10.1371/journal.pone.0170886 (PMC5300236; doi:10.1371/journal.pone.0170886)
Supplement: S1 Fig — (DOC) [file pone.0170886.s001.doc]

**S1 Fig** Scatter plots of correlation coefficients (from (a) to (f)) between the distribution of 10 min midbrain/cerebellum ratios (MID/CE) in [I-123]-ADAM SPECT image, age and body composition in 20 morbidly obese and non-obese young adults

(a)

(b)

(c)

(d)

(e)

(f)
