# Supplementary material for: Comparison of brain serotonin transporter using [I-123]-ADAM between obese and non-obese young adults without an eating disorder
Source: PLoS One. 2017 Feb 9;12(2):e0170886. doi: 10.1371/journal.pone.0170886 (PMC5300236; doi:10.1371/journal.pone.0170886)
Supplement: S1 Table — (DOC) [file pone.0170886.s003.doc]

**S1 Table** Comparisons of SERT in midbrain/cerebellum (MID/CE) ratios between obese and non-obese young adults stratified by sex.

|  | Morbidly Obese | Non-Obese | *p*-value* |
| --- | --- | --- | --- |
| Women |  |  |  |
| Number of cases | 5 | 5 |  |
| Body mass index (kg/m^2^) | 40.60 ± 4.6 | 20.50 ± 0.9 | 0.008 |
| MID/CE (10 min), SERT | 1.39 ± 0.16 | 1.41 ± 0.08 | 0.841 |
| MID/CE (6 h), SERT | 2.57 ± 0.29 | 2.65 ± 0.57 | 0.841 |
| Men |  |  | *p-*value* |
| Number of cases | 5 | 5 |  |
| Body mass index (kg/m2) | 40.00 ± 3.9 | 20.20 ± 1.4 | 0.008 |
| MID/CE (10 min), SERT | 1.34 ± 0.14 | 1.28 ± 0.10 | 1.000 |
| MID/CE (6 h), SERT | 2.42 ± 0.62 | 2.29 ± 0.31 | 0.690 |

*Mann-Whitney test.

SERT, serotonin transporter; MID/CE, midbrain/cerebellum ratios.
